# A mutation in C. neoformans mitochondrial NADH dehydrogenase results in increased virulence in mice

**DOI:** 10.1080/21505594.2020.1831332

**Published:** 2020-10-24

**Authors:** Mitch Merryman, Jacob Crigler, Rebecca Seipelt-Thiemann, Erin McClelland

**Affiliations:** a Department of Biology, Middle Tennessee State University, Murfreesboro, TN, USA; b M&P Associates, Murfreesboro, TN, USA

**Keywords:** *Cryptococcus neoformans*, *Galleria mellonella*, serial passage, NADH dehydrogenase

## Abstract

Cryptococcus neoformans: (H99W) was serially passaged in the invertebrate wax moth *Galleria mellonella* fifteen times to study how fungal virulence evolves under selection and whether those adaptations affect virulence. The *G. mellonella* passaged strain (P15) and the pre-passage H99W strains were used to infect three different host models of *C. neoformans: C. elegans, G. mellonella*, and Balb/c mice. While there was no difference in survival in the invertebrate models, P15 killed mice faster than H99W through both intratracheal and intravenous routes of infection and mice infected intravenously with P15 showed higher fungal burden in the brain. Characterization of the major virulence factors of *C. neoformans* found that P15 had increased capsule size, GXM release, and melanization. Whole genome sequencing of P15 and H99W revealed two mutations in P15, an insertion in the promoter region of NADH dehydrogenase (CNAG_09000) and an insertion in the *LMP1* gene (CNAG_06765). Both ATP production and metabolic rate were higher in P15 compared to H99W. Quantitative RT-PCR suggested that the increased ATP was due to increased RNA levels of NADH dehydrogenase. Thus, adaptation to growth in hemocytes resulted in increased production of ATP, increased metabolic rate, and increased virulence in mice. This was likely due to differential expression of virulence factors, which skewed the host immune response to a less efficient Th2 response, with higher levels of IL-4, IL-10, and TNF-α in the brain. Overall, serial passage experiments have increased our understanding of how this yeast evolves under innate immune selection pressure.

## Introduction

The pathogenic yeast *Cryptococcus neoformans* causes fungal meningitis in patients with compromised immune systems, particularly those infected with HIV. Mortality from cryptococcal infections is extremely high, with reports of 220,000 new cases per year and 181,000 deaths per year [1]. *C. neoformans* has a number of virulence factors it utilizes within the host, such as a polysaccharide capsule, the production of melanin, and the production of urease [2]. One of its essential virulence attributes is its ability to survive and replicate within phagocytic cells of the immune system [3] as intracellular residence allows *C. neoformans* to escape the host immune response and disseminate throughout the host. The complete mechanism of how *C. neoformans* survives within phagocytic cells is not entirely clear; though, it is known that increased capsule size contributes to resistance to oxidative stress [4] and shed capsule promotes permeability of the phagosomal membrane [5], and modulates phagosomal pH [6]. Thus, understanding the mechanism behind intracellular replication may lead to the identification of new microbial factors related to *C. neoformans* pathogenesis and, ultimately, new therapeutics for treatment.

As this yeast primarily causes disease in immunocompromised hosts, there is little data on virulence evolution, though there is evidence that microevolution does occur [7,8], including in the mitochondrial genome [9]. Since the immunocompromised host has an insufficient immune response, it seems likely that evolutionary pressure may be limited and less complex to investigate, but that has not been tested. The use of serial passages gives investigators a powerful method to experimentally evaluate how pathogens adapt and evolve to hosts and has been used to identify factors involved in virulence in a variety of pathogens such as: influenza [10], gallid herpesvirus 2 (which causes Marek’s Disease [11],) and human adenovirus [12]. Passaged strains usually become more virulent in the new host and are attenuated in their original host [13]; one reason why this technique is often used to make vaccines. When strains of *C. neoformans* were serially passaged in mice, they manifested increased virulence, as measured by reduced time to death [14]. Comparison of pre- and post-passaged strains led to the identification of a variety of genes that were previously unknown to be involved in virulence [15]. Thus, depending on the selection pressures (i.e. interaction with certain cells of the host immune system), serial passages can be used to study specific aspects of a host-pathogen interaction in real time.

There are a number of model hosts used to study *C. neoformans* infections and pathogenesis besides mammalian models, such as *Acanthamoeba castellanii* amoeba [16], the fly *Drosophila melanogaster* [17], the nematode *Caenorhabditis elegans* [18], the Tobacco Hornworm caterpillar *Manduca sexta* [19], the silkworm *Bombyx mori* [20] and the waxworm *Galleria mellonella* [21]. *G. mellonella* is an increasingly useful invertebrate host for *C. neoformans*. The larvae are cheap, easy to work with, and have a basic innate immune response consisting primarily of phagocytic hemocytes that engulf and kill *C. neoformans* [21], likely through the production of high levels of reactive oxygen species (ROS) [22]. Additionally, numerous *C. neoformans* virulence factors are important in both *G. mellonella*, as well as mammalian infections [21]. Serial passage has been conducted before in *G. mellonella* using *Aspergillus flavus* [23,24]. The authors found no difference in virulence, but that was likely due to the small number of passages. To date, serial passages in *G. mellonella* have not been performed using *C. neoformans*. To investigate how *C. neoformans* evolved under innate immune selection, the serotype A strain H99W [8] was serially passaged in the wax moth larvae *Galleria mellonella* at 37°C for 100 *C. neoformans* generations. We hypothesized that serial passage of *C. neoformans* in *G. mellonella*, compared to the pre-passaged strain, would result in a *C. neoformans* passaged strain that would be more resistant to ROS and/or have a higher level of intracellular replication.

## Methods

### Passages

*C. neoformans* serotype A strain H99W [8] was grown in Yeast Peptone Dextrose (YPD) broth (Fisher Scientific, Waltham, MA, catalog # DF0428-17-5) from frozen stock at 37°C for 2–3 days and then washed 3 times with phosphate-buffered saline (PBS) and counted. Cells (1 × 10^3^) were suspended in 5 µL PBS and were injected into the last left proleg of 15 larvae of the wax worm, *Galleria mellonella* and then larvae were incubated at 37°C for 3 days. After 3 days, larvae were rinsed in 100% ethanol and the last left proleg was cut with scissors. Hemolymph was collected from each larva, diluted and serial dilutions were plated for colony forming units (CFU) on YPD agar. Non-diluted hemolymph from each larva was pooled and counted. Penicillin/Streptomycin (10,000 U/ml, Invitrogen, Carlsbad, CA, catalog # 15,140,122) was added to the pooled hemolymph and 1 × 10^3^ cells in 5 µL were injected into 15 larvae for the next passage.

Passages were continued until 100 generations of *C. neoformans* had occurred within *G. mellonella*. The doubling time was calculated based on the CFU of the initial inoculum and the average CFU from the 15 larvae from each passage using the following equation: Time infected*(1/Ln(Final CFU/Initial CFU)). The number of generations was calculated by dividing the time infected by the calculated doubling time for each passage.

### G. mellonella *infections*

*C. neoformans* strains H99W and P15 were grown in YPD from frozen stock at 37°C for 2–3 days and then washed three times with PBS and counted. Cells (1 × 10^5^) were suspended in 5 µL PBS and were injected into the last left proleg of 15 larvae of *Galleria mellonella* for each strain. Additionally, 15 larvae were injected with PBS as a control. Larvae were then incubated at 37°C and monitored daily for death or pupation.

### C. elegans *killing assay*

*C. neoformans* strains H99W and P15 were grown in YPD from frozen stock at 37°C for 2–3 days and then washed three times with PBS and counted. Cells (~1 × 10^9^) of each strain were plated in a lawn onto nematode growth medium agar and grown overnight at 30°C. The next day, ~25 wild type (strain N2) *C. elegans* adult worms were placed on each plate (5 plates/*C. neoformans* strain) and incubated at 25°C. Worms were monitored every 12 h for 6 days for death.

### Mouse infections

All animal use complied with federal regulations and the University of Scranton Institutional Animal Care and Use Committee guidelines. The protocol was approved by the Committee on the Ethics of Animal Experiments of the University of Scranton (protocol #2-08).

Four–six week-old female Balb/c mice (NCI, Charles River) were injected either intravenously or intratracheally with 1 × 10^6^ cells of strain H99W, P15 or PBS. For intravenous infections, 100 µL of each strain was injected directly into the tail vein. For intratracheal infections, mice were injected intraperitoneally with a 2.5:1 mix of ketamine:xylazine to anesthetize them (5–10 mg/kg, Butler Schein, Miami Lakes, FL, catalog #s 045822 and 033197, respectively) prior to surgery. For the procedure, mice were placed on their back, their neck area was cleaned with alcohol, and a small incision was made over the thyroid. The skin was gently pulled aside and 50 µL of each strain was injected directly into the trachea using a bent tuberculin needle. The incision was closed using VetBond (Fisher Scientific, catalog #NC9259532) and the mice were kept warm and observed closely until they regained consciousness.

For survival experiments, mice were observed over the course of infection. Any mouse in a moribund state and/or distress was euthanized to avoid unnecessary suffering. For CFU experiments, mice were euthanized at day 7 post-infection by CO_2_ inhalation and spleen, lungs, brain, and blood were collected. Blood was centrifuged at 10,000 × *g* for 2 minutes, the sera was removed and stored at −80°C. Organs were homogenized in PBS plus proteinase inhibitors against serine, cysteine and metalloproteases (Roche, Complete Mini, Basel, Switzerland, catalog #11,836,153,001) using a tissue homogenizer. Two hundred microliters of organ homogenate was diluted and plated for CFU counts while the remaining homogenate was centrifuged at 4580 × *g* for 10 minutes to remove cell debris. Organ supernatant was collected and stored at −80°C until use.

### Mouse Cytokine Enzyme-Linked Immunosorbent Assays (ELISAs)

BD-OptEA kits (BD Biosciences, San Jose, CA) were used to assay organ supernatants for levels of interleukin (IL)-4, IL-10, IL-12, tumor necrosis factor (TNF)-α and interferon (IFN)-γ, per the manufacturer’s instructions. Organ homogenate (from CFU experiments) was tested without dilution for every mouse in each infection group. The detection limits of the cytokine assays were 7.8 pg/mL for IL-4, 31.3 pg/mL for IL-10, 62.5 pg/mL for IL-12, 15.6 pg/mL for TNF-α, and 31.3 pg/mL for IFN-γ, as stated by the manufacturer.

Phenotypic Analysis. For all of the following phenotype analyses, P15 and H99W were grown in YPD from frozen stock at 37°C for 2–3 days and then washed three times with PBS and counted.

### Capsule Size

Capsule size *in vitro* was measured as described [25] and repeated twice. Briefly, 1 × 10^5^ cells/mL of H99W and P15 were added to Dulbecco’s modified Eagle medium (DMEM, Invitrogen, catalog # 10,566–016) and incubated at 37°C + 10% CO_2_ for 18 h. Cells were collected, suspended in 10 μL PBS and added to a microscope slide with India Ink (Fisher Scientific, catalog # 14–910-56). Cells were imaged on an Eclipse TS100 Nikon microscope (Tokyo, Japan) with a 100× objective. For each strain, pictures of 30 cells were taken. The diameter of the cell body and capsule were measured using Nikon Elements software. Capsule diameter was calculated by subtracting the cell body diameter from the diameter of the entire cell + capsule and dividing by 2.

### Glucuronoxylomannan (GXM) Release

To determine if the strains differed in their ability to release capsular GXM into the medium, capsules were induced in DMEM (as for measuring capsule size). The next day, the DMEM supernatant was collected and the concentration of GXM in the media was measured by capture ELISA as described [26]. This experiment was repeated three times.

### Melanin Production

*C. neoformans* strains H99W and P15 were grown in YPD from frozen stock at 37°C for 18–24 h (log-phase) and then washed three times with PBS and counted. Cells (1 × 10^6^ cells/mL) were inoculated into minimal media containing L-3,4-dihydroxyphenylalanine (L-Dopa, Sigma Aldrich, St. Louis, MO, catalog #D1507) and incubated at 30°C, shaking for 7 days. Melanization was assessed by absorbance of the suspension at 400 nm on days 1, 3, 5 and 7. If a strain produced melanin, the cells turned a brown/black color. This experiment was repeated three times.

### Urease Activity

The urease activity of the passaged strains was determined as described [27]. Briefly, 5 × 10^7^ cells were suspended 1:1 with PBS and 2X Roberts Urea broth and incubated at 37°C for 4 h. After 4 h, the strains were centrifuged to pellet the cells and the supernatant was measured by spectrophotometry at 560 nm on an M5 spectrophotometer (Molecular Devices, San Jose, CA). Urease production was indicated by pink color in the supernatant. This experiment was repeated three times.

### Intracellular Survival in Macrophages

To determine how well strains H99W and P15 survived in macrophages, intracellular fungal burden was determined as described [15]. Briefly, J774.16 macrophages-like cells were seeded into a 96-well plate in DMEM containing 10% FCS (Invitrogen, catalog # 10,437–028), 10% NCTC-109 (Invitrogen, catalog # 21,340–039), 1% non-essential amino acids (Invitrogen, catalog # 11,140–050) and 100 U/ml IFN-γ (Roche, catalog # 11,276,905,001) at a density of 5 × 10^5^ macrophages/mL and incubated overnight at 37°C with 5% CO_2_. *C. neoformans* strains H99W and P15 were grown for 18–24 h (log phase) in YPD media at 37°C, washed with three times with PBS, counted and opsonized at 37°C for 30 minutes with the capsular monoclonal antibody 18B7 (a kind gift from Dr. Arturo Casadevall). H99W and P15 were added in a 1:1 ratio of macrophages to fungi in DMEM with 1 μg/mL lipopolysaccharide (Sigma Aldrich, catalog # L3137) and 200 U/mL IFN-γ to a concentration of 2 × 10^5^/mL and incubated at 37°C with 5% CO_2_ for 1 h. After 1 h, extracellular *C. neoformans* were removed by gentle washing 5× with warm PBS. The J774.16 cells with phagocytized *C. neoformans* were then resuspended with warm DMEM and incubated for 24 h at 37°C + 5% CO_2_. The next day, J774 cells were lysed by adding 100 µL 0.5% SDS to the media. After 5 min, the wells were washed 2× with 100 µL PBS and the media plus all washes were pooled, diluted and plated on YPD (two plates per well) for 2 days at 37°C, after which colonies were counted to determine fungal burden. This experiment was repeated four times.

## Genome sequencing

Genomic DNA was isolated from passaged strains P15 and H99W using the Gentra Puregene Yeast Kit (Qiagen, Hilden, Germany, catalog #158567) with an excess of lytic enzyme solution and RNase followed by column purification using the Blood and Cell Culture DNA Mini Kit (Qiagen, Genomic Tip 20/G, catalog #10223). Once purified, genomic DNA was electrophoresed on an agarose gel to determine its size and integrity. Then, genomic DNA was cleaned with a Genomic DNA Clean & Concentrator™-10 column (Zymo Research, Irvine, CA, catalog #gDCC-10) and quantified with a Qubit BR DNA assay (ThermoFisher Scientific, catalog # Q32850). Nextera XT libraries (Illumina, San Diego, CA, catalog # FC-131-1024) were made using 0.5 ng input DNA. Libraries were quantified with a Qubit HS DNA assay, (ThermoFisher Scientific, catalog # Q32854) manually normalized, and denatured with 0.1 N NaOH. A MiSeq run was done with paired-end 250 bp reads using a V2 500 cycle reagent kit (Illumina, catalog # MS-102-2003).

## Next-Generation Sequencing Strain Genome Analysis

Illumina paired end reads for the parent (H99W) and the evolved strain P15 were generated using the MiSeq platform (Lubbock, TX). The parent strain library (H99W) had a total of 960,868 paired sequences. The *Galleria*-evolved strain library (P15) had a total of 539,377 paired sequences. The sequencing reads were checked for quality using FastQC [28] and trimmed of adapters and low quality reads using a 20:20 sliding window using trimmomatic [29]. Greater than 99.5% of paired reads survived trimming. The trimmed data for each strain was aligned to the concatenated *Cryptococcus neoformans* strain H99 reference genome (NC_026745.1) using bowtie2 [30]. Greater than 98% of paired reads aligned to the genome. Samtools was used to convert the resulting .sam files to a .bam files, index the resulting .bam data files, and generate variant statistics for all nucleotide positions based on an indexed genome file, which was also generated using samtools [31]. Bcftools [31] was then used to generate the variant call files based on the variant statistics. The indexed genome sequence file, gene annotation file for *Cryptococcus neoformans* strain H99S, indexed .bam file, and the variant call file were co-visualized using the Integrated Genome Viewer [32] to inspect depth of coverage at variant call positions for each strain. Variant calls with quality scores equating to less than 1:100,000 (QUAL<50) or coverage depth less than 10 were not included. The confirmed variant positions were then compared between the strains. Five variants were present in the *Galleria*-evolved strain that were not also present in H99W or a lab evolved strain (data not shown). The sequencing data from this project has been deposited in the NCBI BioProject Sequence Read Archive (SRA Accession ID: SUB4386347).

## ATP production

ATP production was measured in cell supernatants as described in [33]. Briefly, *C. neoformans* strains H99W and P15 were grown for 18–24 h (log phase) in YPD media at 37°C, washed 3× with PBS, and counted. A total of 5 × 10^8^ cells were frozen at −80°C overnight. The next day, the cells were resuspended in a 4:1 ratio of 50 mM HEPES buffer (Sigma Aldrich, pH = 7.75) and dimethyl sulfoxide (Sigma Aldrich) and homogenized in a bead beater 5× for 45 sec (on ice for 45 sec in between). Cell lysates were centrifuged at 1500 × *g* to pellet cell debris and the total protein concentrations of the supernatants were determined using a BCA Protein Concentration assay (Pierce, Appleton, WI, catalog #23235). ATP production was measured using an ATP Determination Kit (Molecular Probes, Eugene, OR, catalog #A22066), per the manufacturer’s instructions. Measurements were done in triplicate for each strain. ATP concentrations were calculated using a standard curve for every assay. This experiment was repeated three times.

## 2,3-Bis-(2-Methoxy-4-Nitro-5-Sulfophenyl)-2 H-Tetrazolium-5-Carboxanilide (XTT) assays

H99W and P15 cells were grown to mid-log phase at 37°C, washed 3× with PBS, and diluted with PBS. Cells (5 × 10^7^ cells/mL) were diluted in minimal media in a 96-well plate (4–6 wells per strain), incubated for 20 h at 37°C, and then XTT (Sigma Aldrich, 1 mg/mL) and Menadione (Sigma Aldrich, 1 mM in acetone) were added to each well. The plate was incubated for a further 4 h at 37°C, centrifuged at 750 × *g* for 1 min and the supernatant transferred to new wells. The absorbance was measured at 492 nm. This experiment was conducted three times.

## Quantitative Reverse Transcriptase PCR (qRT-PCR)

We isolated total RNA from H99W or the evolved P15 strain grown to log-phase in YPD. Total RNA was extracted (RNAeasy Kit, Qiagen, catalog #75142) and genomic DNA was removed (Message Clean Kit, GenHunter, Nashville, TN, catalog #M601). cDNA was reverse transcribed from RNA (Quantitech Reverse Transcription kit, Qiagen, catalog #205310), the integrity of the RNA was assessed using the Qubit 2.0 fluorometer (ThermoFisher Scientific) and real-time quantitative PCR was performed using Power up SYBR Green (ThermoFisher Scientific, catalog #A25742), and primers specific to *CNAG_09000* (NADH dehydrogenase subunit 1). The fold change relative to the pre-passage H99W strain was calculated using the geometric mean of three different housekeeping genes (*ACT1, TFC1* and *UBC6*) as the control gene [34]. The fold change for each transcript was calculated relative to the pre-passage H99W strain. qRT-PCR was repeated twice with three different biological replicates.

### Statistics

Capsule diameter measurements, cytokine levels and CFU were analyzed using the nonparametric Wilcoxon Rank Sums test, while the amount of GXM released was analyzed using analysis of variance (ANOVA). ATP production was analyzed using MANOVA with simple contrasts while differences in metabolic rate were analyzed using ANOVA. Melanin production was analyzed using linear regression. Survival was analyzed using the Kaplan-Meier test. *p* < 0.05 was considered significant.

## Results

### Strain generation

**Figure 1.**
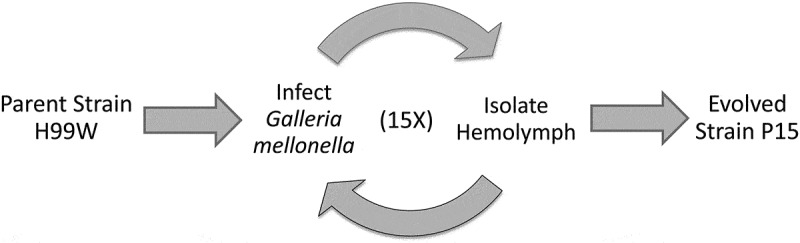
Schema of strain generation

To generate a strain of *C. neoformans* that evolved under innate immune selection, the serotype A, H99W strain [8] was passaged in *G. mellonella* (P15) 15 times, ~100 *C. neoformans* generations (Figure 1).

### Virulence studies

**Figure 2.**
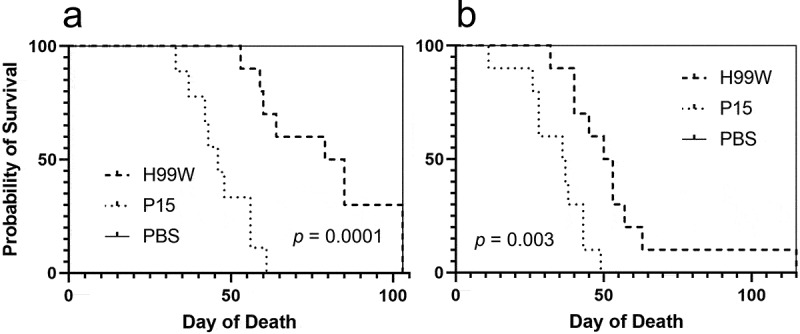
Survival data of mice infected with P15 or H99W. (a) Mice infected intratracheally with strain P15 show decreased survival compared to mice infected with the pre-passage strain H99W or the PBS control. Ten mice were infected for each group. (b) Mice infected intravenously with strain P15 show decreased survival compared to mice infected with the pre-passage strain H99W or the PBS control. Ten mice were infected for each group

**Figure 3.**
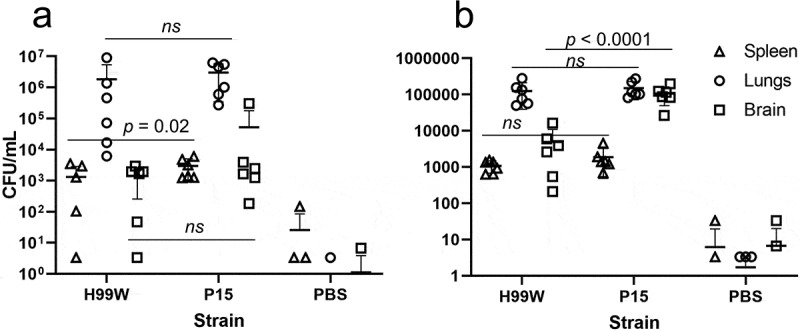
Fungal burden in mice. (a) Mice infected intratracheally with strain P15 have higher fungal burden in the spleen, but there is no difference in fungal burden between mice infected with strain H99W or P15 in the lungs or the brain. Error bars represent the standard deviation of the mean. Six mice are infected in each group. Zero values are not plotted for PBS. (b) Mice infected intravenously with strain P15 show increased fungal burden compared to mice infected with the pre-passage strain H99W or PBS. Error bars represent the standard deviation of the mean. Six mice are infected in each group. Zero values are not plotted for PBS

To determine whether the passaged strain (P15) had evolved increased virulence due to innate immune selection after passage in *G. mellonella*, strains H99W and P15 were used to infect three host models with different immune responses: the roundworm *C. elegans, G. mellonella*, and Balb/c mice. While P15 and H99W killed *C. elegans* and *G. mellonella* similarly (data not shown), P15 killed mice significantly faster than H99W, through both intratracheal (Figure 2(a), *p* = 0.0001) and intravenous (Figure 2(b), *p* = 0.003) routes of infection. While mice infected intratracheally with strain P15 had significantly higher fungal burden in the spleen, there was no difference in fungal burden in the lungs or brain at day 7 post-infection (Figure 3(a), *p* = 0.02). In contrast, mice infected intravenously with P15 had significantly higher brain fungal burden (Figure 3(b), *p* < 0.0001) at day 7 post-infection, suggesting that, in this model of infection, increased fungal burden contributed to the increased death of mice.

### Phenotypic analysis of passaged strains

**Figure 4.**
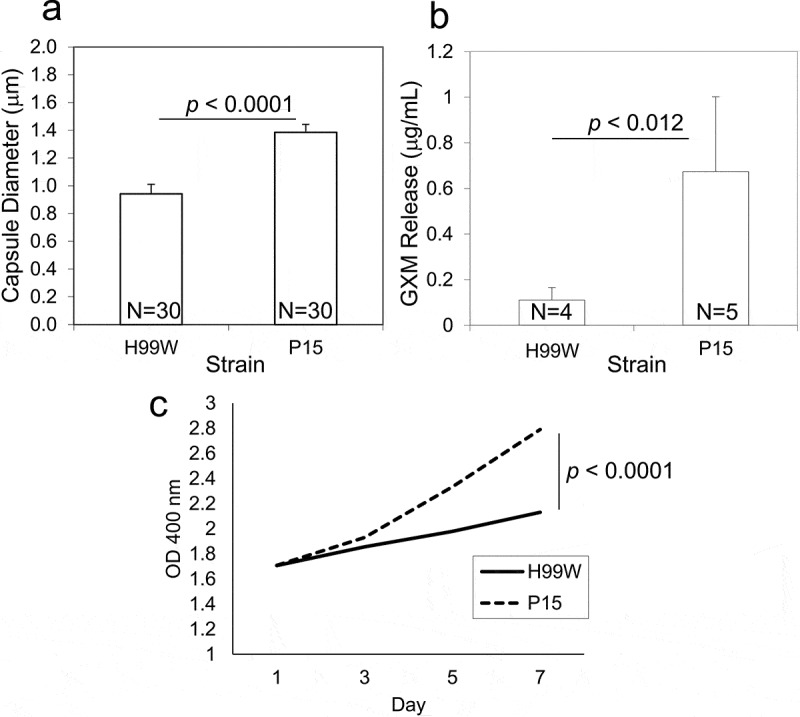
Capsule size, GXM release and melanin production is increased in strain P15. (a) Bar graph showing increased capsule diameter (µm) in strain P15 compared to the pre-passage strain H99W. The graph is a representative graph of two independent experiments. Error bars represent the standard error of the mean. Numbers within bars are the numbers of cells measured per strain. (b) Bar graph showing increased GXM release in strain P15 compared to the pre-passage strain H99W. The graph depicts the average of two independent experiments. Error bars represent the standard deviation of the mean. Numbers within bars represent the sample size. (c) Line graph showing increased melanization of P15 compared to H99W. The graph depicts the average of three independent experiments

To further investigate the underlying mechanism of observed differences in virulence, the passaged strain was characterized for changes in known virulence factors. After 100 generations in *G. mellonella*, the capsule diameter of *C. neoformans* strain P15 was significantly increased compared to the pre-passage H99W strain (Figure 4(a), *p* < 0.001). Additionally, strain P15 released significantly more GXM (Figure 4(b), *p* < 0.01), and made more melanin than the pre-passage H99W strain (Figure 4(c), *p* < 0.0001), likely contributing to its increased virulence in mice. There were no differences in phospholipase B or urease production, doubling time in YPD, phagocytic index, or fungal burden in J774.16 macrophages (data not shown).

### Genome sequencing & analysis

To identify mutations in the P15 genome that might be responsible for the increased virulence in mice, the genomes of the passaged strain P15 and H99W were sequenced. Sequence analysis revealed five mutations in P15 compared to H99W: 1) a 27 bp insertion in the promoter region of the gene encoding mitochondrial NADH dehydrogenase (subunit 1 of the electron transport chain, *CNAG_09000*), 2) a 7 bp insertion in the protein coding region of exon 1 of *LMP1* (low mating performance, *CNAG_06765* [8],), 3) a 1 bp deletion in the intergenic region between *CNAG_07784* and *CNAG*_*07785*, both of which encode hypothetical proteins, 4) a substitution in intron 2 of *CNAG*_*01001*, which encodes a hypothetical protein, and 5) a substitution in a non-genic region at the end of chromosome 1, past the 3ʹ end of *CNAG_07934* (Table 1).

**Table 1.** Mutations observed in the P15 strain not found in the parent strain

Chromosome	Nearest Gene(s)	Chromosomal position (nt)	Reference Sequence	Mutant Sequence	Mutation Type	Predicted Effect
CnMt	CNAG_09000	2	TTA	TTATATCATTGTATTGAGTACCTCTGATTA	Insertion	Regulatory
Cn02	CNAG_06765	99,354	A	AACTGGCC	Insertion	Shifts reading frame at codon 142 which truncates the protein at codon 155
Cn09	CNAG_07784 and CNAG_07785	1,160,375	AC	A	Deletion	Intergenic, likely no effect
Cn05	CNAG_01001	1,465,552	A	G	Substitution	Intronic, likely no effect, but possibly regulatory
Cn01	CNAG_07394	2,287,370	C	G	Substitution	Intergenic, likely no effect, but possibly regulatory

**Table 2.** qRT-PCR data showing that P15 likely has slightly higher levels of NADH dehydrogenase subunit 1 (*CNAG_09000*) RNA relative to H99W

Experiment number	Fold Change of Strain H99W	Fold Change of Strain P15	Biological Replicate #
1	1.0	1.12	Set 1
1	1.0	1.43	Set 2
1	1.0	2.27	Set 3
2	1.0	2.22	Set 1
2	1.0	1.03	Set 2
2	1.0	1.00	Set 3

### ATP and XTT assays

**Figure 5.**
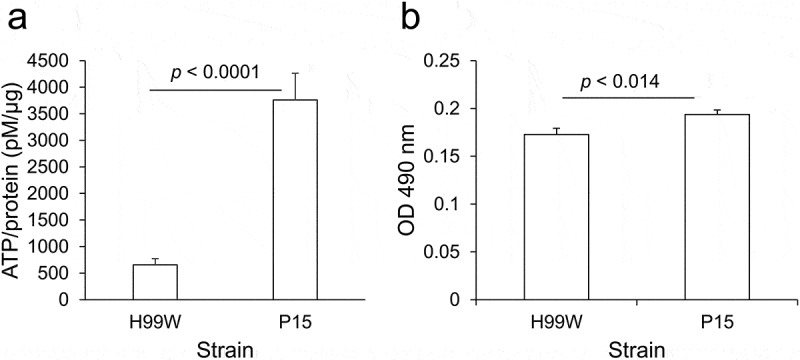
ATP production and metabolism is increased in strain P15 compared to H99W. (a) P15 makes more ATP than the pre-passage H99W parent strain. The graph is representative of three independent experiments. Error bars represent the standard deviation of the mean. (b) P15 has an increased metabolic rate compared to H99W, as measured by XTT reduction assay, in minimal media. The graph is representative of three independent experiments. Error bars represent the standard error of the mean

A mutation of the promoter encoding the NADH dehydrogenase gene might play a role in the increased virulence by affecting energy availability and enabling robust fungal growth. Thus, for each strain, the amount of ATP produced and the metabolic rate, via XTT reduction assay, which measures the activity of several dehydrogenases present in active mitochondria [35], were measured. P15 produced 5.7-fold higher amounts of ATP than H99W (Figure 5(a)) and had a higher metabolic rate than H99W (Figure 5(b)), suggesting that P15 exhibited higher levels of respiration and metabolism than H99W.

### qRT-PCR

The identification of the NADH dehydrogenase promoter insertion in strain P15 combined with the increased ATP levels and metabolic rates for strain P15 suggested that the mutation led to higher ATP levels by increasing steady state levels of NADH dehydrogenase RNA. qRT-PCR was performed to quantify the level of NADH dehydrogenase RNA in the passaged strain compared to the H99W pre-passage strain. P15 had a 1.5-fold average increase in RNA of NADH dehydrogenase compared to H99W (Table 2), suggesting this could be the reason for the increased production of ATP.

### Mouse Immune response

**Figure 6.**
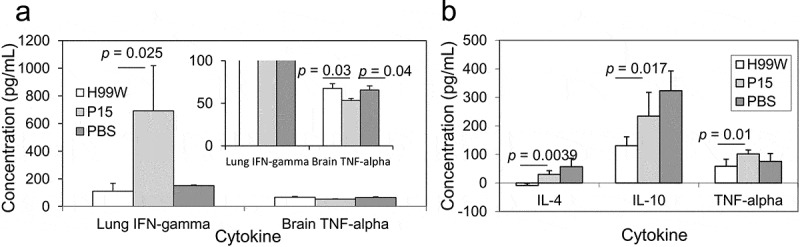
Cytokine production after intratracheal (a) and intravenous (b) infection with *C. neoformans*. (a) IFN-γ production in lungs of mice and TNF-α production in the brain of mice. Mice infected intratracheally with strain P15 show increased production of IFN-γ in the lungs, but decreased production of TNF-α in the brain compared to mice infected with the pre-passage strain H99W or PBS. White bars represent mice infected with H99W, light gray bars represent mice infected with strain P15, and dark gray bars represent mice infected with PBS. Error bars represent the standard error of the mean. Six mice were infected for each group. Significance was tested using a Wilcoxon Rank Sums test and ANOVA, respectively. (b) Mice infected intravenously with strain P15 show increased production of IL-4, IL-10, and TNF-α in the brain compared to mice infected with the pre-passage strain H99W. White bars represent mice infected with H99W, light gray bars represent mice infected with strain P15, and dark gray bars represent mice infected with PBS. Error bars represent the standard deviation of the mean. Six mice were infected for each group. Significance was tested using a Wilcoxon Rank Sums test

Because strain P15 was more virulent in mice compared to the pre-passage H99W strain, cytokine profiles in the different host organs were measured to characterize the host immune response. Mice infected intratracheally with strain P15 had a significantly higher production of IFN-γ in the lungs at day 7 post-infection (*p* = 0.025), but significantly lower production of TNF-α in the brain at day 7 post-infection (*p* = 0.038) compared to mice infected with H99W (Figure 6(a)), suggesting a complex host response. In contrast, mice infected intravenously with P15 had significantly higher levels of the cytokines IL-4 (*p* = 0.0039), IL-10 (*p* = 0.017) and TNF-α (*p* = 0.01) in the brain at day 7 post-infection compared to mice infected with H99W (Figure 6(b)), suggesting the increased virulence in this route of infection was due to an increased Th2 response in mice.

## Discussion

To better understand what factors enable *C. neoformans* to persist and replicate intracellularly, serial passage through the waxworm *G. mellonella* was used to create an evolved strain, P15. We hypothesized that serial passage of *C. neoformans* within hemocytes of *G. mellonella* would result in a strain that had increased intracellular replication, ultimately resulting in a strain with increased virulence [13].

To test that hypothesis, three model hosts with different immune responses were infected: *C. elegans, G. mellonella*, as well as Balb/c mice with H99W and P15 and differences in host survival were determined. While there was no difference in survival between strain P15 and H99W in *C. elegans* and *G. mellonella*, there was a difference in *in vivo* fungal burden in *G. mellonella* (Ali et al., 2020), suggesting that the serial passages resulted in increased virulence. It is unclear why there was no difference in survival in *C. elegans*. The original manuscript describing this model noted that increased capsule size was not required for killing of *C. elegans* [18], so another virulence factor besides the capsule may be important for pathogenesis in this model. Further studies are needed to delineate this. In contrast, there was a significant difference in survival in mice, both by intravenous and intratracheal routes of infection (Figure 2(a-b)). In addition, mice infected intravenously had higher fungal burdens in the brain (Figure 3), which is evidence of increased tissue replication and suggests an ineffective host immune response. Thus, serial passage of *C. neoformans* in *G. mellonella* resulted in a strain adapted to survive within *G. mellonella* hemocytes, and those adaptations translated to increased fungal burden and virulence in mice.

Serial passage of *C. neoformans* in the wax moth larvae *G. mellonella* for 100 *C. neoformans* generations also resulted in definitive phenotypic changes in the yeast. The passaged strain P15 had a larger polysaccharide capsule, released more GXM, and made more melanin than the pre-passaged strain (Figure 5(a–c)). There are a number of known conditions that result in increased capsule size in *C. neoformans*, including exposure to CO_2_ [36], growth in limited amounts of iron [37], growth in conditions of limiting nutrients which slow cell growth [38], and growth in the presence of ROS [4]. Given that the passaged strain was likely exposed to both limiting nutrients and ROS within *G. mellonella*, this may explain the increased capsule size seen in the passaged strain, especially since capsule enlargement has been shown to increase resistance to ROS [4]. The fact that strain P15 also released more GXM is not surprising, given that GXM has been shown to act as an antioxidant that protects cells against damage caused by ROS [4]. Strain P15 also made more melanin than H99W, likely because melanin acts to scavenge free radicals and thus plays a protective role in stress resistance [39]. These phenotypic changes also most likely contributed to the increased fungal burden and virulence observed in mice.

To determine if there might be specific mutations that could account for the increased virulence of strain P15, the genomes of P15 and H99W were sequenced. Five mutations were observed in P15, but only two were considered to be possibly linked to the hypervirulence observed in mice: 1) an AT-rich insertion in the promoter region of NADH dehydrogenase subunit 1, and 2) an insertion in the *LMP1* gene (*CNAG_06765*). NADH dehydrogenase subunit 1 is part of complex 1 of the electron transport chain [40], while the *LMP1* gene has been implicated in mating and virulence [8]. While there was no difference in mating between P15 and H99W (data not shown), as *LMP1* is involved in virulence in a mouse model [8] and is involved in titan cell formation [41], it is possible that this mutation contributes to the virulent phenotype of P15.

Because NADH dehydrogenase (subunit 1) is part of complex I in the electron transport chain, it was possible that the insertion in the promoter region of this mitochondrial gene affected ATP production and metabolism in P15. P15 produced 5.7-fold more ATP (Figure 5, *p* < 0.0001) and had a higher metabolic rate (Figure 5(b), *p* < 0.014) than H99W. Thus, these data may explain the observed decrease in survival, as in various bacteria, changes in metabolism after colonization of a new niche have been linked to increases in virulence [42]. Additionally, the pathogenic yeast form of the fungus *Talaromyces marneffei* has 3–7-fold higher levels of TCA cycle intermediates compared to the nonpathogenic hyphal form, which affects yeast growth in THP-1 macrophages, suggesting that increased metabolism affects growth and virulence [43]. This has also been observed in *C. albicans*, where a mutant with increased respiration forms biofilms better than the wildtype strain [44].

The NADH dehydrogenase insertion itself is AT-rich (TATCATTGTATTGAGTACCTCTGATTA) and within 20 bp of the transcription start site, which has been shown to increase promoter activity in nuclear promoters of *S. cerevisiae* [45], although the effect of adding AT-rich elementals to cryptococcal mitochondrial promoters has not been investigated. To determine whether the increase in the amount of ATP produced was due to an increase in the amount of steady state RNA encoding NADH dehydrogenase, quantitative RT-PCR was used to quantify NADH dehydrogenase subunit 1 RNA levels between the strains. P15 showed a 1.5-fold average increase in NADH dehydrogenase RNA levels compared to H99W. We believe this increase is biologically relevant, as it would lead to an increase in ATP, but not too much ATP produced, which has negative consequences for protein expression in the cell [46]. In the intracellular environment of a hemocyte, gaining a mutation that increases RNA levels of NADH dehydrogenase subunit 1 would be very advantageous since it is known that oxidants and RNS inhibit complex I of the electron transport chain [47,48]. This would be especially beneficial if *C. neoformans* produces polygenic mitochondrial RNAs, as is seen in *S. cerevisiae* [49]. Since many of the genes required for complex I are mitochondrially encoded and reside near the gene encoding NADH dehydrogense, the overall result could be increased expression of many of the genes encoding complex I and increased production of ATP. Thus, this insertion was likely crucial for P15 to adapt to continued growth within hemocytes and the sustained presence of ROS.

*G. mellonella* has an innate immune response comprised of fungistatic fatty acids located in the cuticle, phenoloxidases that instigate the formation of melanin, antimicrobial peptides that inhibit various microbes (mostly bacterial) and hemocytes that contain ROS and encapsulate invading microbes to form granulomas (nodules) around the microbe so that the microbes can be lysed [50]. In contrast, the mammalian immune response is much more complex and is comprised of both an innate immune response, as well as a specific adaptive immune response. The mammalian innate immune response includes antimicrobial peptides, neutrophils, and monocytes that produce both ROS and RNS to kill any engulfed microbes, as well as complement and natural killer cells to mark and destroy invading microbes. The mammalian adaptive immune response employs B lymphocytes to produce antibodies and activated T lymphocytes that are specific for the invading microbe and mark it for destruction [51]. Thus, given that the murine immune response is much more complex than that of *G. mellonella*, it is surprising that a strain of *C. neoformans* that was passaged in *G. mellonella* is so virulent in mice. This may be due to increased production of ATP, increased metabolic rate, as well as the increased release of GXM in strain P15, as GXM has been shown to inhibit activation of alveolar macrophages, resulting in less production of ROS [52].

To determine how the increased virulence of P15 affected the host immune response, mice were infected with strains P15 and H99W and cytokine profiles were determined at day 7 post-infection. Mice infected intratracheally showed a significant increase in production of IFN-γ in the lungs of mice infected with strain P15, but a significant decrease in TNF-α in the brain, suggesting stimulation of the inflammatory immune response in the lungs, but not the brain, indicating a complex host response. A possible reason for the increased virulence in this route of infection is that proinflammatory damage in the lungs may play a role in the increased virulence of strain P15 in mice; however, this hypothesis needs to be tested in the future. Since IFN-γ is required for activation of alveolar macrophages [53], this suggests that in the lungs, strain P15 may activate macrophages to stimulate an immune response. Unfortunately, given the survival data of mice infected intratracheally and the decreased levels of TNF-α in the brain, the stimulation of the lung proinflammatory immune response was not sufficient to clear the infection. Given these contradictory findings, further studies are needed to determine the mechanism for increased virulence in this infection model.

For the intravenous infection, mice infected with strain P15 had significantly higher production in the brain of the Th2 cytokines IL-4 and IL-10, as well as the inflammatory cytokine TNF-α. The Th2 response in the brain is likely responsible for the increased fungal burden seen in the brain, which is not surprising given that a Th2 response has been shown to be a permissive environment for cryptococcal growth [54]. Taken together, these data suggest that while mouse alveolar macrophages may try to control the infection by stimulating an inflammatory response in the lung, once in the brain, the increased release of GXM by strain P15 biased the immune response to a nonproductive Th2 response. The end result was significantly increased death in mice, which is not unexpected as GXM has been shown to inhibit a Th1 response by inhibiting inflammatory cytokines [55].

In summary, serial passage of *C. neoformans* in *G. mellonella* resulted in a strain with increased virulence in a mouse model and different virulence factor phenotypes. We hypothesized that serial passage of *C. neoformans* in *G. mellonella* would result in increased virulence due to increased intracellular replication. While increased virulence was observed, increased intracellular replication (at least in J774.16 macrophages) was not observed, suggesting that the increased virulence in mice was due primarily to the phenotypic changes in strain P15 virulence factors, increased ATP production, and increased metabolic rate, which negatively affected the host immune response. This research highlights the use of serial passages to study microbe-host interactions in real time and has increased the understanding of how *C. neoformans* evolves in the presence of the innate immune response of *G. mellonella*.

## Data Availability

The genome sequences of H99W and P15 have been deposited in the NCBI BioProject Sequence Read Archive (SRA Accession ID: SUB4386347). https://www.ncbi.nlm.nih.gov/sra/SUB4386347

## Abbreviations

P15: strain from passage 15
GXM: glucuronoxylomannan
ROS: reactive oxygen species
RNS: reactive nitrogen species
YPD: yeast peptone dextrose
CFU: colony forming unit
PBS: phosphate-buffered saline
DMEM: Dulbecco’s Modified Eagle Medium
FCS: fetal calf serum
IFN-γ: interferon gamma
TNF-α: tumor necrosis factor alpha
IL-4: interleukin 4
IL-10: interleukin 10
